# Abstract of a Paper on Veterinary Dentistry1Read at the August meeting of the Onedia County Veterinary Medical Association.

**Published:** 1896-10

**Authors:** R. C. Hurlburt

**Affiliations:** Boonville, N. Y.


### ABSTRACT OF A PAPER ON VETERINARY DENTISTRY.[1]

By R. C. HURLBURT, D.V.S.,
BOONVILLE, N. Y.

In presenting this paper I shall only refer to a few salient points on this important branch of our work. Many facts come to us by experience that are not heard from the lecture desk or found in text-books. I consider that this branch has not received the attention its importance warrants, even among our best patrons, when we are awakened by the appearance of a travelling dentist with a showy kit of tools, and who takes more with him than has been our portion for a long time, and leaves the impression that we are not equipped for this work. Often his careless work, when viewed from his predictions as to the great results to follow, add much to the owner's lack of

[1] Read at the August meeting of the Onedia County Veterinary Medical Association.

confidence in the whole subject of dentistry. Veterinary dentistry does not require a specialist. A knowledge of anatomy and the mechanism of these organs of digestion must be understood. There can be no fixed rules for this work, but judgment and care are essential in every case dealt with. The work of the teeth means constant change, and this suggests the importance of watchful care over these organs, that they may conserve the best interests of the animals which depend so largely upon them for growth and development. Floating or dressing the teeth for the removal of irregularities on the edges and corners constitutes the chief work. Those of the external border of the superior and internal borders of the inferior molar teeth are chiefly the parts requiring our attention. The edges should be dressed but little lower than the table surfaces of the teeth. At times the first upper molar and the last inferior molar of the same side may have projections, and the reverse condition is less often met with where the lower first molar extends beyond the superior one of the same side, and the reverse condition of the opposing sixth molars of the same side. These are fruitful of trouble in perfect mastication and demand care and skill in their adjusting.

The rapid wear of the deciduous teeth when young animals are being conditioned for severe work is at times surprising. The hard food given to mature the system causes deep points and projections to rapidly appear, and, the tongue and cheeks becoming lacerated, these animals are difficult to train. The smallness of the mouth and its condition ofttimes require the exercise of special skill and kindness to rectify the altered condition. Removal of deciduous teeth is frequently requisite.

The removal of permanent teeth, broken or diseased, is a matter of great difficulty at times, particularly when the fifth or sixth lower are involved.

---

A Cheap Education. The Wisconsin Eclectic Medical College, an incorporated institution of the "Badger State," offers a cheap medical education. Among the inducements offered is the following, which is taken from a letter recently received by a veterinarian: "No matter what the difficulty may be, financial or otherwise, let us hear. We will, however, anticipate financial trouble, and, feeling disposed toward you, are willing to make yours a special case, and will reduce our total fees to $25.00."

